# Editorial: Combat sports in contemporary society: an interdisciplinary exploration

**DOI:** 10.3389/fspor.2025.1568909

**Published:** 2025-02-12

**Authors:** Lazar Toskić

**Affiliations:** ^1^ Faculty of Sport and Physical Education, University of Priština-Kosovska Mitrovica, Leposavić, Serbia; ^2^ Faculty of Sport, University ‘’Union-Nikola Tesla’’, Belgrade, Serbia

**Keywords:** combat sports, technique, physiology, psychology, biomechanics, performance, socio-culture

**Editorial on the Research Topic**
Combat sports in contemporary society: an interdisciplinary exploration

Combat sports have come a long way from traditional martial arts, whose main practical purposes were self-defence and warfare, to the type of physical activity that is included in every cultural and educational aspect of sport. Today, as their social and health benefits have been well recognised, combat sports are an indispensable part of the sports scene, with thousands of competitors, globally reputed athletes of high impact, and grand competitions supported by marketing and sponsorship. Furthermore, combat sports are part of the physical education curriculum in schools and training systems for socially important, security-related professions (police, military etc.), allowing for the adequate growth and development of children and youth, mastery of self-defence skills, and ensuring personal and public safety. Finally, combat sports are a popular recreational activity with a constant increase in participants.

The special issue of *Combat sports in contemporary society: an interdisciplinary exploration*’ aims to bridge the fields of physiological, psychological, sociological, and technological studies. The majority of the published articles explore the technique, biomechanics and performance issues in various combat sports. Santos et al. investigated the Body Acceleration (BA) profile in a judo contest in the male and female weight divisions. Subjects participating in a 5-min simulated match contest against a same-sex opponent from the same weight division wore an accelerometer to record heart rate, blood lactate and ratings of perceived exertion. The study results revealed the differences in the athletes’ BA and three distinct profiles were identified, suggesting that the demands placed on judo athletes in a contest differ across weight divisions and sexes. Another study by Xu et al. aimed to determine the differences in kinematic parameters associated with cross and uppercut punches between Sanda athletes and Boxing athletes and to analyse their impacts on peak punching speed. The punches from both groups of athletes were compared in terms of 13 key parameters utilizing a three-dimensional framework and high-speed cameras. The results revealed significant differences in six cross-related parameters and four uppercut-related parameters. Incognito et al. examined the role of the opponent's head in predicting the target of a kicking action in martial arts. A sample of mixed combat athletes and non-athletes watched a series of video clips depicting various kicking techniques with differing levels of spatial occlusion of the head, with no significant effect of expertise on accuracy. Head occlusion did not significantly influence performance nor did it interact with expertise, suggesting that head and face information did not play a role in predicting opponent action intent.

Another aspect of combat sports extensively investigated in this special issue is physiological. A group of authors (Sek et al.) conducted a study aimed to determine the weight loss methods used before an official championship and their effects on the performance of wrestlers. Data from the sample of 350 competitive wrestlers were collected using the Athlete Weight Loss Methodology and Effects Scale and a personal information form. It was determined that wrestlers preferred weight-loss methods such as restricting food and fluids, using a sauna, and jogging with a raincoat. The mini review by Levy et al. also explores the psychological factors associated with weight-cutting practices among combat sports athletes. The authors concluded that implementing gradual weight loss strategies in combat sports may offer numerous advantages, but that education of the sport staff is necessary. One study (Nema et al.) dealt with both performance and physiological parameters in combat sports. The objective of this study was to assess the relationship between selected performance indicators of aerobic and anaerobic capacity to sports performance in elite karatekas. The Karate Specific Aerobic Test and the Wingate Test were applied to measure aerobic and anaerobic endurance while technical and tactical indicators were used to assess the level of athletic skill during competition. The high predictive validity confirmed the importance of a high level of anaerobic conditions for performance in karate.

Psychology, as an important factor in sport success, is also covered in this special issue. Neuropsychological impact of Sanda training on athletes’ attention was investigated by Teng et al. The Attention Network Test was administered to a sample of professional Sanda athletes and a control group to compare differences in the efficiency of the alerting, orienting, and executive control networks. Compared to the control group, the Sanda athletes exhibited significant efficiency in the higher executive control network and the executive control network. Additionally, Piepiora et al. in their study emphasised the importance of mental preparation for kata competitors in karate, processing the specificity and methods of mental training.

Finally, the special issue includes studies that were concerned with the socio-cultural aspect of combat sports. Li et al. investigated gender equality, that is, the involvement of Chinese women in combat sports. Not only did the authors reveal that Chinese women still faced numerous restrictions in combat sports, but they also presented the factors which may lead to this phenomenon and proposed several possible solutions for the problem. Authors Cheng and Guo implemented the educational ethnography method to explore curriculum content construction to ensure the inheritance of martial arts as intangible cultural heritage at universities. In the process, the emphasis is placed on local knowledge, core skills, cultural traditions, and other characteristics that highlight the excellence of the cultural heritage of martial arts. Santanna and Li conducted the study that explores the formation of a hybrid body image among white cisgender males practicing Wushu at the Siberian Chinese Martial Arts Center, in which narrative interviews with 12 participants were conducted and three main themes were revealed by thematic analysis.

Hopefully, the presented studies will contribute to further the understanding and development of combat sports and prove useful for future research in this field.

